# In vitro comparison of cleaning efficacy and force of cylindric interdental brush versus an interdental rubber pick

**DOI:** 10.1186/s12903-021-01558-4

**Published:** 2021-04-14

**Authors:** Christian Graetz, Kristina Schoepke, Johanna Rabe, Susanne Schorr, Antje Geiken, David Christofzik, Thomas Rinder, Christof E. Dörfer, Sonja Sälzer

**Affiliations:** 1 Clinic of Conservative Dentistry and Periodontology, University of Kiel, Arnold-Heller-Str. 3, Haus B, 24105 Kiel, Germany; 2 Institute of Mechatronics, Computer Science and Electrical Engineering, Kiel University of Applied Sciences, Kiel, Germany

**Keywords:** Oral hygiene, Interdental brushes, Mechanical plaque control, Interdental cleaning efficacy

## Abstract

**Background:**

Interdental brushes (IDB) are according to the actual evidence the first choice for cleaning interdental areas (IDR). Their size should be chosen individually according to the IDR morphology. However, interdental rubber picks (IRP) are appreciated better by the patients and are hence becoming more and more popular but the evidence regarding their efficacy is still limited. The aim of this in vitro study was to measure the experimental cleaning efficacy (ECE) and force (ECF) during the use of interdental brushes versus newer wireless types with rubber filaments (IRP), both fitted and non-fitted for different IDR.

**Methods:**

The medium size of a conical IRP (regular, ISO 2) with elastomeric fingers versus four sizes (ISO 1, 2, 3, 4) of cylindric IDB with nylon filaments (all Sunstar Suisse SA, Etoy, Switzerland) were tested. Interdental tooth surfaces were reproduced by a 3D-printer (Form 2, Formlabs Sommerville, MA, USA) according to human teeth and matched to morphologically equivalent pairs (isosceles triangle, concave, convex) fitting to three different gap sizes (1.0 mm, 1.1 mm, 1.3 mm). The pre-/post brushing situations at IDR (standardized, computer aided ten cycles) were photographically recorded and quantified by digital image subtraction to calculate ECE [%]. ECF were registered with a load cell [N].

**Results:**

Overall, a higher ECE was recorded for IDB compared to IRP (58.3 ± 14.9% versus 18.4 ± 10.1%; *p* < 0.001). ECE significantly depended on the fitting of the IDB. ECE was significant higher in isosceles triangle compared to concave and convex IDR for both IDB and IRP (*p* ≤ 0.001). ECF was lower for IDB (0.6 ± 0.4N) compared to IRP (0.8 ± 0.5N; *p* ≤ 0.001). ECE in relation to ECF increases with smaller IDB. For IRP highest values of ECF were found in the smallest IDR.

**Conclusions:**

Within the limitations of an in vitro study, size fitted IDB cleaned more effectively at lower forces compared to conical IRP.

## Background

In the last decades, the focus in dentistry has shifted from intervention to prevention, so it’s not surprising that optimizing oral hygiene continues to be a central aspect in daily routine. However, as up to date the bristles of tooth brushes do not reach the interproximal surfaces of teeth efficiently [1], additional devices are necessary to penetrate between adjacent teeth [2]. Diverse cleaning devices are available. Among them dental floss, tooth picks, and interdental brushes are the most commonly used worldwide. However, only low-certainty evidence exists regarding the cleaning efficacy of these cleaning aids [3] and data for newer devices like interdental rubber picks (IRPs) are inconsistent [4, 5].

It is common sense that interdental brushes should be individually chosen according to the morphology of the interdental areas (IDR) [6] in order to clean effectively without inducing any hard tissue abrasion or soft tissue trauma. However, there is no evidence regarding application and correct choice of size [7]. Furthermore, IRPs are a relatively newly developed devices with an increasing market but only little evidence [8]. On the other hand, they are highly accepted by patients [9].

In a previously published investigation, our primary aim was to develop a new experimental setup to test in vitro*,* under standardized, controlled, and reproducible conditions, the interdental experimental cleaning efficacy (ECE) and the cleaning force of different types of rubber picks [10]. This was necessary, as the currently available clinical measurements lack the absolute validity and reliability to assess the interdental cleaning efficacy in vivo [11] and on the other hand, the majority of in vitro studies are neglecting more clinically relevant morphologies of the interdental areas, such as convex or concave shapes of the proximal root surfaces [12].

The aim of the present in vitro study using our reproducible experimental setup, was to compare the experimental cleaning efficacy and the cleaning force between different sizes of interdental brushes (ISO 1, 2, 3, 4) versus a standard interdental rubber pick (regular, ISO 2) for different interdental areas. Our primary hypothesis was, that cleaning with interdental brushes would lead to higher experimental cleaning efficacy when compared to the interdental rubber pick. Furthermore, we suppose that these effects would be significantly influenced by the size of the cleaning aid.

## Methods

### Experimental setup

**Fig. 1:**
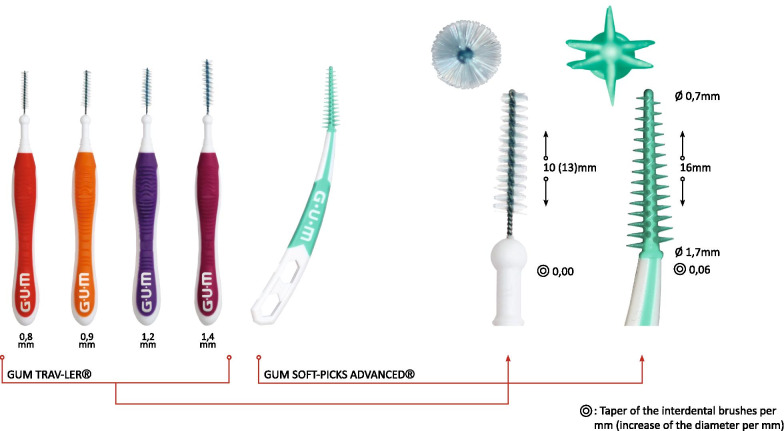
Illustration of the test devices for interdental cleaning (from the left): interdental brushes (IDB) with nylon with a wire core and nylon filaments with diameter of 0.8, 0.9, 1.2, 1.4 mm (ISO 1–4) and wireless interdental rubber picks (IRP) with finger-design (magnification showed in detail the different design of interdental brushes and rubber picks). The working part of the IRP is 16 mm with a taper of 0.05 of the core. The IDB shows no taper having a cylindrical shape, but a working part of 10 mm

In this in vitro study, the medium size of conical IRP with a diameter of 0.7 mm increasing to 1.7 mm resulting in a taper of 0.06 (regular, ISO 2; GUM SOFT-PICKS® Advanced, Sunstar Suisse SA, Etoy, Switzerland) was tested versus IDB with a wire core and nylon filaments with diameter of 0.8, 0.9, 1.2, 1.4 mm (ISO 1–4; Trav-ler, Sunstar Suisse SA, Etoy, Switzerland) as illustrated in Fig. 1. As explained in detail in our previous publication [10], we used a computer software (Autodesk Fusion 360, Autodesk Direct Limited, Hampshire, United Kingdom) and in vivo data of interdental morphologies [12–14] to design and print 3D composite replicas in stereolithography manner (Form 2, Formlabs Sommerville, MA, USA) by using liquid photopolymer resin (White Resin V04 (RS-F2-GPWH-04), Formlabs, Sommerville, MA, USA). This experimental setup was originally developed, in order to achieve the highest possible reproducibility and accuracy [15]. To simulate the interdental cleaning process, the replicas were fixed pairwise in a socket with an embedded load cell (KD34s, ME-Meßsysteme GmbH Hennigsdorf, Germany; measuring range: ± 500 mN with precision class of 0.1%). This allowed a continuous measuring of the applied forces during ten cleaning cycles and an automatic documentation in a table (Microsoft Excel 2016, Microsoft Corporation, Redmond, WA, USA), as well as the removal and replacement of the adjacent teeth surfaces in a reproducible manner. Due to the background noise of the load cell between two cleaning cycles, only data > 0.09N were included.

**Fig. 2:**
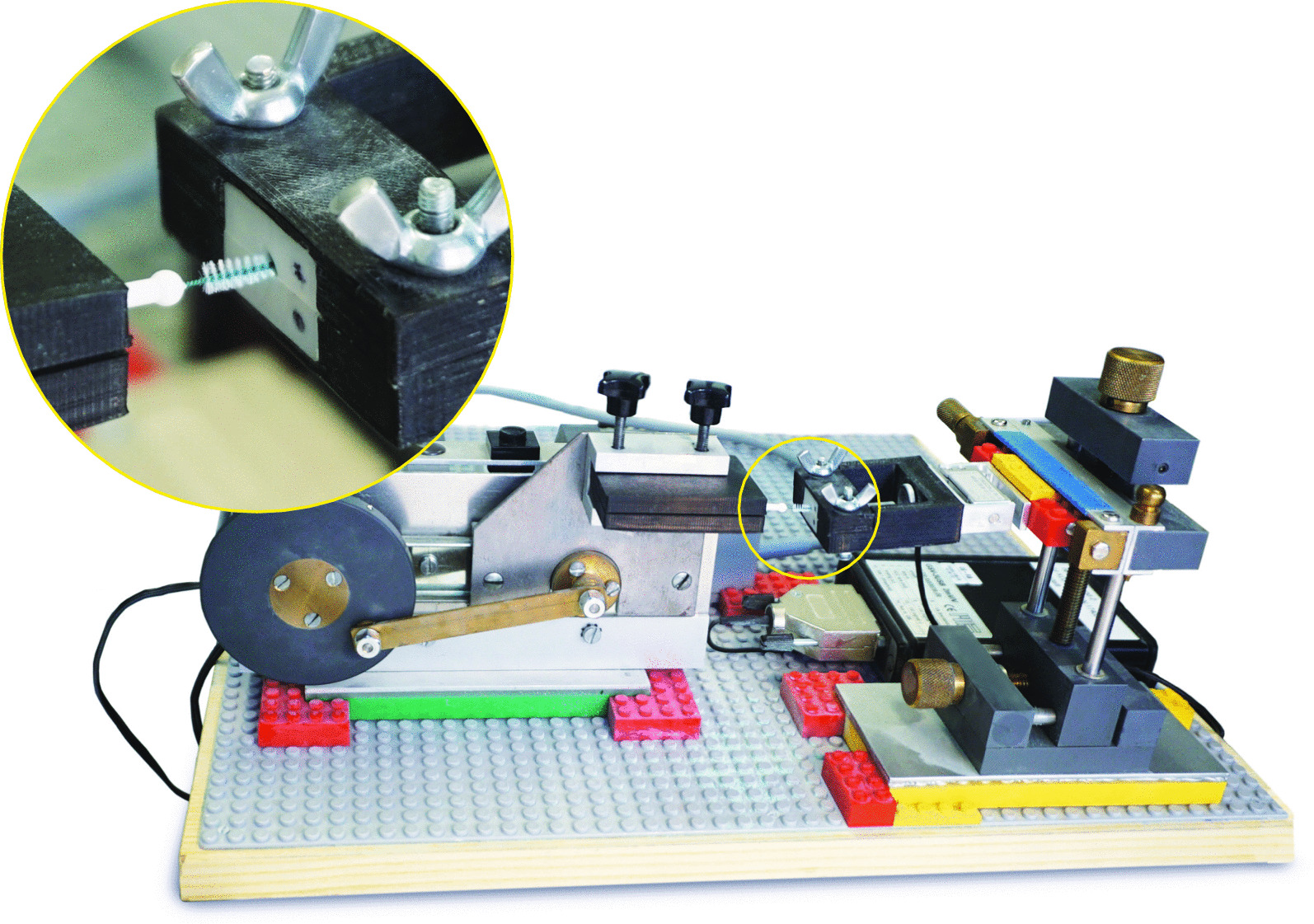
Overview of experimental setup mechanical device, which converts rotation into linear motion moves the test products into the different artificial interdental areas. The insert on the upper right corner illustrates the four different morphologies of artificial interdental areas (from left: isosceles triangle, concave space of 3 mm height, concave space of 5 mm height and convex space; all shown morphologic in size 1.3 mm. The digital load cell records the applied force longitudinally and documents it in a table chronological (not shown), control unit for motion and load cell and the electric transformer

We used three interdental gap sizes of 1.0 mm (small), 1.1 mm (medium) and 1.3 mm (large) in four morphologies (isosceles triangle, convex, concave space of 3–5 mm height), resulting in nine different artificial interdental areas. Subsequently, the interdental area replicas were stained by one investigator (K.S.) with Occlu Spray Plus (Hager & Werke, Duisburg, Germany) as described in previous studies [10, 16, 17]. A standardized powder thickness (mean ± SD: 20 ± 5 µm) was ensured by a standardized procedure and appropriate time protocol. The baseline surface was digitally photographed (Canon EOS 400D Digital, Uxbridge, United Kingdom) and documented. Afterwards, a mechanical device, which converts rotation into a horizontal motion, moved the interdental cleaning aids with a controlled speed ten times (10 × for- and back-ward) into the artificial interdental area (Fig. 2). After the test, all artificial interdental area replicas were again photographed in order to subsequently perform an evaluation of ECE by digital image subtraction (Image J, NIH, Bethesda, USA). Testing in a reproducible manner was proofed [10].

ECE was determined as the difference of simulated biofilm before and after cleaning the interdental area in percent. ECF, the force (in Newton) for cleaning the IDR was calculated as the average force value (mean ± SD) of ten cycles pushing and pulling the test products inside the artificial IDR.

Conclusively, ECE was related to ECF with a superiority of ECE.

### Image analysis

The images were extracted via XN View to enhance the most important area of the interdental space. Afterwards the picture section was edited by Photoshop in the same procedure as described in detail in our first study [10] to get a digital image subtraction (ImageJ, NIH, Bethesda, MD, USA). The cleaned areas have a different color in contrast to non-cleaned areas.

### Statistical analysis

A power calculation for the determination of the sample size was based on the results of a previously published in vitro study on the cleaning efficacy (percent of removed simulated biofilm) and resistance to insertion of two different interdental brushes [17]. According to this sample size calculation (sub-group analysis was considered beforehand), we found n = 25 samples per group as sufficient to detect five percent difference for experimental cleaning efficacy between the groups of different test products with a power of 80%.

For statistical analysis, data were entered in SPSS Statistics (SPSS Statistics 24, IBM, Chicago, IL, USA). Mean values of the ECE and of the ECF were calculated for every tested product and type/gap size of artificial interdental area separately. Normal distribution of the recorded values was tested with the Kolmogorov–Smirnov/Lilliefors test. For all data there was no normal distribution (*p* < 0.001). Subsequently, a mean value comparison was performed using the non-parametric Mann–Whitney-U-test and Kruskal–Wallis-test. A linear regression assessed associations between predictors (type/size of interdental area, type/size of test product) and ECE (dependent variable). Regression coefficients, standard errors (SE), *p* values and 95% confidence intervals (CI) were used as effect estimates. Association of ECE and ECF was evaluated using Pearson’s correlation coefficient and between ECE and IDR type and size with Spearman/Eta-Coefficient. All tests were two-sided. Statistical significance was assumed if *p* ≤ 0.05.

## Results

An overview for ECE and ECF measurements is given in Table 1. It should be noted, that we failed in 49 out of 900 tests performed to analyze ECE (ECF: 28 out of 900 tests) and the data could not be used in the final assessment.

**Table 1:** Subgroup results (mean ± SD) of experimental cleaning efficacy (ECE in %) and experimental cleaning forces (ECF in N) of all test products

	IDR 1.0 mm			IDR 1.1 mm			IDR 1.3 mm		
	Isosceles triangle	Convex	Concave	Isosceles triangle	Convex	Concave 3 mm/5 mm	Isosceles triangle	Convex	Concave
*Experimental cleaning efficacy (ECE in %)*									
IDB ISO 1 0.8 mm	81.7 ± 3.6	40.5 ± 2.8	53.3 ± 10.8	n.a	n.a	n.a	n.a	n.a	n.a
IDB ISO 2 0.9 mm	84.2 ± 3.5	44.9 ± 2.4*	60.8 ± 10.5	76.9 ± 4.1	48.5 ± 2.8	53.8 ± 13.6	77.8 ± 5.3	47.8 ± 2.1*	48.3 ± 10.3*
IDB ISO 3 1.2 mm	n.a	n.a	n.a	n.a	n.a	43.7 ± 3.8*	79.6 ± 3.7	55.3 ± 2.1*	55.9 ± 11.2*
IDB ISO 4 1.4 mm	n.a	n.a	n.a	n.a	n.a	54.3 ± 2.5	n.a	n.a	61.1 ± 2.8****
IRP ISO 2 0.9–1.0 mm	41.6 ± 4.2*	13.9 ± 1.5*	14.8 ± 3.3*	29.1 ± 7.4	12.2 ± 1.7*	11.3 ± 1.7*	28.7 ± 5.3*	10.2 ± 2.3*	15.1 ± 3.8*
*Force for cleaning (ECF in N)*									
IDB ISO 1 0.8 mm	0.4 ± 0.1*	0.2 ± 0.0*	0.3 ± 0.0*	n.a	n.a	n.a	n.a	n.a	n.a
IDB ISO 2 0.9 mm	0.7 ± 0.1*	0.4 ± 0.1*	0.5 ± 0.1*	0.7 ± 0.1*	0.4 ± 0.1	0.4 ± 0.1*	0.5 ± 0.0*	0.3 ± 0.0*	0.4 ± 0.1*
IDB ISO 3 1.2 mm	n.a	n.a	n.a	n.a	n.a	0.8 ± 0.1***	1.2 ± 0.1*	0.9 ± 0.1*	0.8 ± 0.2*
IDB ISO 4 1.4 mm	n.a	n.a	n.a	n.a	n.a	1.9 ± 0.4*	n.a	n.a	1.5 ± 0.2*
IRP ISO 2 0.9–1.0 mm	1.4 ± 0.2*	0.7 ± 0.1*	1.4 ± 0.3*	0.8 ± 0.1*	0.7 ± 0.2*	0.8 ± 0.4*	0.3 ± 0.1*	0.4 ± 0.2*	0.6 ± 0.4*

Force during ten cleaning cycles (mean ± SD) for cleaning different types (isosceles triangle, convex, concave) and sizes (1.0 mm, 1.1 mm, 1.3 mm) of the interdental area separated for the tested interdental brushes (IDB) versus interdental rubber picks (IRP). We assumed *p* < 0.05 (in bold) to be statistically significant (Mann–Whitney-U-test, Kruskal–Wallis-test, two sided)

*Significant difference to all other tested products

**Significant difference to IDB 0.9 and IRP

***Significant difference to all tested sizes of IDB

****Significant difference to all tested products except for IDB ISO 4

### Cleaning efficacy

Overall, the ECE (mean ± SD) was higher for IDBs (n = 564) compared to IRP (n = 287) (58.3 ± 14.9; range 33.6–90.2 vs. 18.4 ± 10.1%; range 5.6–52.4; *p* < 0.001).

**Fig. 3:**
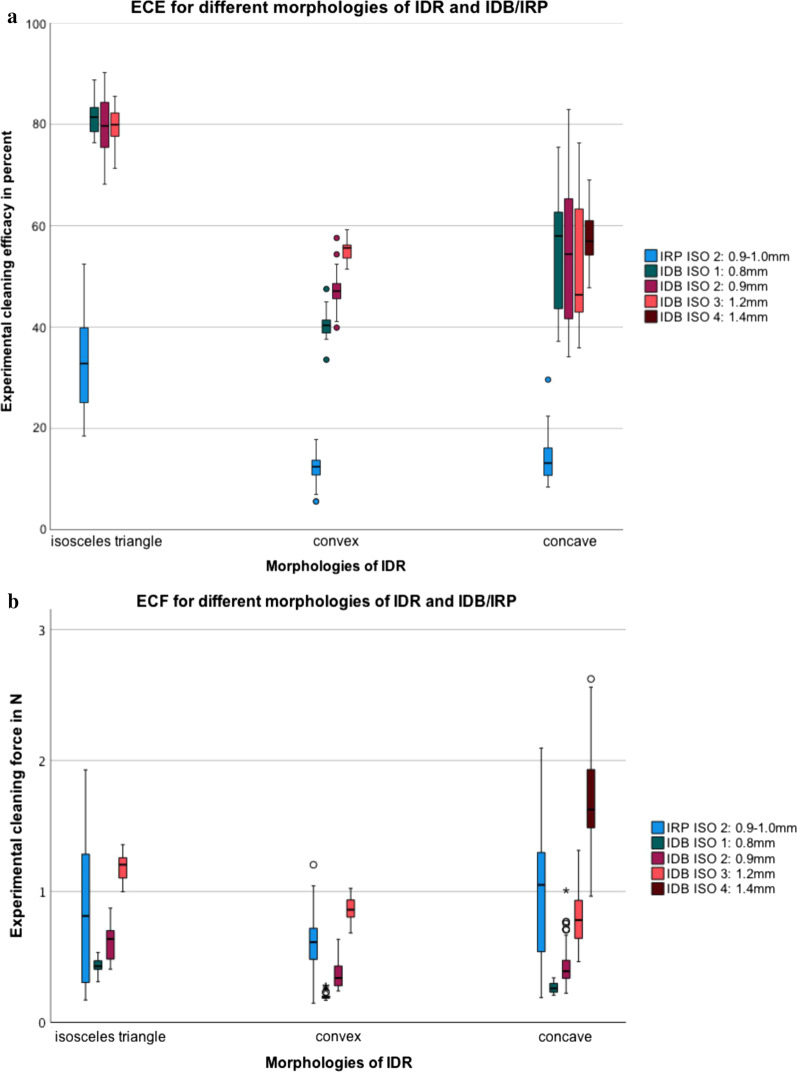
Illustration of **a** the experimental cleaning efficacy (ECE) and **b** the experimental cleaning force (ECF) for different morphologies of interdental region (IDR) and IDB/IRP. Three different IDR on the x-scale (isosceles triangle, convex, concave), the ECE on the y-scale, differing in IRP (blue), IDB ISO 1 (green), IDB ISO 2 dark red, IDB ISO 3 bright red and IDB ISO 4 in brown

Furthermore, ECE was higher in isosceles triangle compared to convex and concave areas for both IDB (80.0 ± 4.8 vs. 47.4 ± 5.5 vs. 54.0 ± 11.2%; all *p* < 0.001) and IRP (33.2 ± 8.3 vs. 12.1 ± 2.4 and 13.7 ± 3.5%; *p* ≤ 0.001 and *p* = 0.114; Fig. 3a). The results for ECE differed significantly between all IDRs morphologies (*p* ≤ 0.001) with one exception between convex and concave areas for IRP (*p* = 0.114). The highest mean ECE was achieved using ISO2 (0.9 mm) IDBs in a 1 mm isosceles triangle (84.2 ± 3.5%) (Table 1).

### Subgroup analysis: efficacy dependent on IDR

**Fig. 4:**
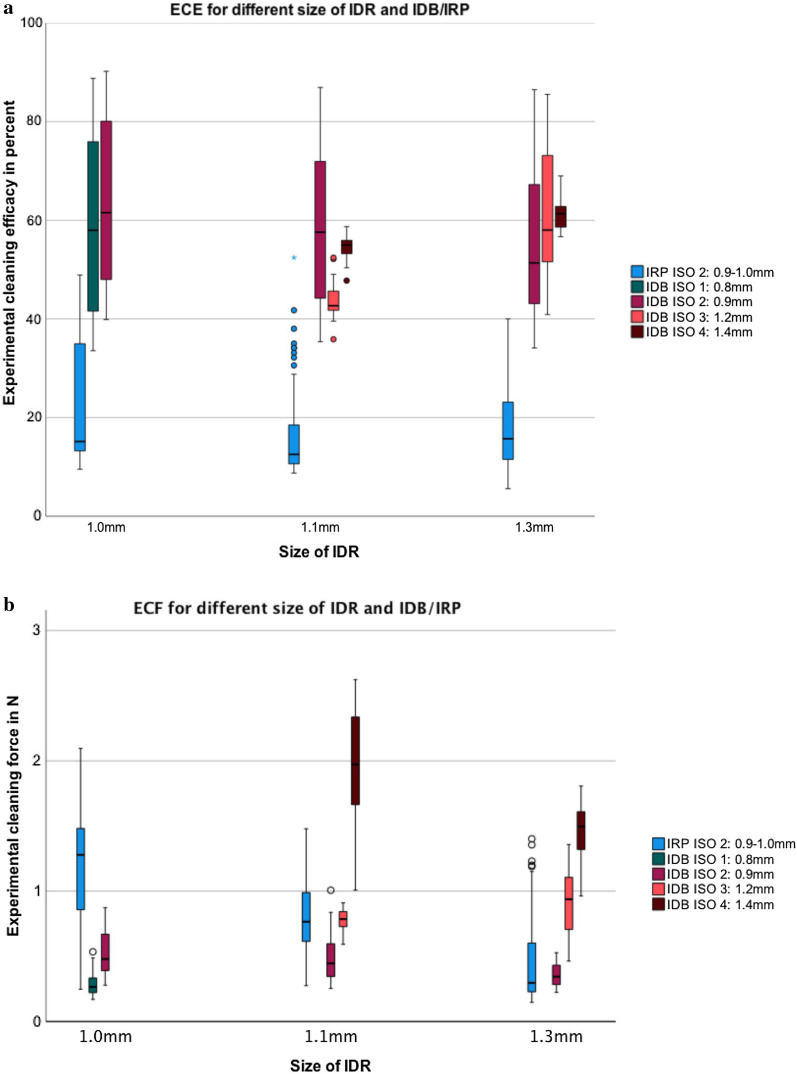
Illustration of **a** the experimental cleaning efficacy (ECE) and **b** the experimental cleaning force (ECF) for different sizes of IDR and IDB/IRP. Three different IDR on the x-scale (1.0 mm; 1.1 mm; 1.3 mm), the ECE on the y-scale, differing in IRP (blue), IDB ISO 1 (green), IDB ISO 2 dark red, IDB ISO 3 bright red and IDB ISO 4 in brown

An interdental room of 1 mm was cleaned with the highest ECE using IDB ISO 2 independent of its shape (Fig. 4a). For isosceles triangle or convex 1.1 mm IDR, the IDB of ISO 2 was best fitted, providing the best cleaning results. In concave shaped IDRs, ISO 4 (1.4 mm) showed the best overall ECE (54.3 ± 2.5%) reaching values up to 61.1 ± 2.8% for a 1.3 mm concave IDR. In convex shaped IDRs, the IDB of ISO 3 (1.2 mm) performed best (55.3 ± 2.1%) and in an isosceles triangle shaped IDR the IDB of ISO 2 and ISO 3 performed equivalently (77.8 ± 5.3%) and 79.6 ± 3.7%). All result and corresponding significant difference for subgroup analysis are shown in Table 1.

### Experimental cleaning forces (ECF)

On average, ECF (mean ± SD) was lower for IDB (0.6 ± 0.4N) compared to IRP (0.8 ± 0.5N; *p* ≤ 0.001). Forces needed with IDB depended on the brush size and its correlation to the space area (Table 1; Figs. 3b, 4b). The highest ECF was found in isosceles triangle compared to convex and concave areas for IDB (0.7 ± 0.3N vs. 0.4 ± 0.2N vs. 0.7 ± 0.5N; all *p* < 0.001 with Bonferroni adjustment). For IRP, no statistical difference was found for the ECF between isosceles triangle (0.8 ± 0.5N) and concave (0.9 ± 0.5N; *p* = 0.557) but cleaning both IDR needed significant higher forces compared to convex morphologies (vs. 0.6 ± 0.2N; *p* ≤ 0.001 with Bonferroni adjustment).

Furthermore, the ECF differed significantly only between IRP sizes 1.0 mm and 1.1 mm (1.0 mm vs. 1.1 mm vs. 1.3 mm: 1.2 ± 0.4N vs. 0.8 ± 0.3N vs. 0.5 ± 0.4N; *p* ≤ 0.001; *p* = 0.065; *p* = 0.070, all with Bonferroni adjustment). For IDB, the cleaning force for IDR size 1.1 mm (0.8 ± 0.6N) was higher compared to 1.0 mm (0.4 ± 0.2N; *p* = 0.035 with Bonferroni adjustment and 1.3 mm (0.7 ± 0.4N; *p* = 0.039 with Bonferroni adjustment) but there was no difference between 1.0 mm vs. 1.3 mm (*p* = 1.000 with Bonferroni adjustment).

The highest mean ECF was registered for IDB ISO 4 (1.4 mm) in a 1.1 mm concave interdental space (1.9 ± 0.4N). For IRP the ECF was highest in the smallest interdental space, in particular in the isosceles triangle 1 mm (1.4 ± 0.2N) and concave 1 mm area (1.4 ± 0.3N).

## Discussion

With the help of our in vitro procedure, it could be demonstrated that IDB provide a significantly better overall cleaning efficacy compared to IRP. Thus, our primary hypothesis was confirmed. The superiority of conical, cylindrical and waist-shaped IDB compared to conical sticks in parallel walled blocks has already been shown in a previous study [18]. The high amount of elastic nylon bristles of IDB might be able to adapt better to the tooth surfaces compared to the fewer elastic fingers of the tested IRP. A further reason for the overall better results for ECE and lower ECF with IDB might be that the artificial interdental area sizes (1.0, 1.1, 1.3 mm) were cleaned with four different sizes of IDB (0.8, 0.9, 1.2, 1.4 mm), but only one size of IRP (conical 0.7 mm increasing to 1.7 mm) was available. In order to confirm our primary hypotheses, the aim was to compare a fitted IDB to corresponding fitted IRP with regard to ECE and ECF (IRP regular versus IDB ISO 2). Secondary aim was to test the importance of fitting IDB regarding different sizes and IDR, which was done by the four sizes of IDB. In our previous study [10] we already tested different sizes of IRP. For an IDR of 1.0 mm, the tested IDB ISO 2 and the IRP (regular, ISO 2) have both a fitted size corresponding to the gap size of the IDR. However, for all shapes of the IDR, ECE is significantly lower for IRP compared to the IDB ISO 2 (Table 1). Besides the lower ECE of IRP, ECF was significantly higher for all 1.0 mm IDR. The thin nylon filament of the tested IDB bends with a smaller resistance than an elastomeric rubber finger of the IRP, especially in small interdental spaces. It must be further assumed, that the rough surface of the IRP, especially in contact with the artificial tooth surface simulated in our study (~ 25 µm vs. ~ 10 µm of natural enamel [19]), deforms more and creates a greater resistance under usage than the surface of the smooth nylon filaments [10].

To understand the cleaning efficacy of IDBs, not only the material, length or diameter [20] of the IDB has to be considered but also the morphology of the interdental space. The sub-analysis of different morphologies of artificial interdental areas demonstrated a difference between IRP and IDB in all different types of interdental space. The highest ECE was measured in isosceles triangle type spaces. Therefore, it was the best-cleaned IDR of all tested interdental spaces. Furthermore, it’s important to consider the relative dimension of size of the IDB in relation to the artificial interdental space [12, 21]. A higher contact area between the IDB and the tooth surface results in an increase of efficacy of cleaning [12], correlating with a higher force application. Therefore, we could prove our previously published hypothesis [10] that the ECF measured for parallel shaped IDB remains constant in a more parallel-walled interdental area, whereas in an equilateral triangular shaped interdental area, the necessary force will increase more with greater IDB dimensions. The overall higher ECF measured for IRP (Table 1) can be explained by the higher contact area between (1) the rubber fingers and the tooth surface (lower elastic and wider diameter compared with nylon bristle of IDB), (2) a higher coefficient of friction for silicon on the composite of the IDR replicas as well as (2) the higher taper (conical type compared with the parallel shaped IDB).

Hence, for the choice of the best suited IDB, its diameter should fit adequately in order to achieve the best possible ECE but not be too large to avoid possible trauma concomitant with higher ECF. However, it remains to be clarified which force is needed to cause a clinical trauma. It is shown that the ECF increases faster than the ECE decreases using “non-fitted” IDB [13].

In our in vitro study, we tested the IRP in each interdental space, although they were not fitted to larger IDR. This corresponds to the use of “non-fitted” IRP (or IDB) by non-instructed users. Furthermore, we have to assume that IDBs were used only a maximum of 2–3 sizes at home. Therefore, in a real-life situation, the applied IRP/IDB is not always the best-fitted device for each individual morphology [3]. Consequently, as an alternative to “fitted” IDB, it might be better to use IRP to minimize any hard tissue abrasion or soft tissue trauma due to higher forces of “non-fitted” IDB, possible traumas caused by the wire, and the risk of causing abrasions to the gingiva using oversized brushes (Table 1). Future in vitro-studies should investigate if different IRP sizes will improve the ECE in comparison to IDB.

Although IRP still won’t be the first choice concerning the cleaning efficacy, they show good results as a supplement. The advantage of IRP is their wireless construction—they show high primary stability without bending or fracturing of the core in this and our previous test [10]. IRP are more and more promoted and developed, and could be seen as the next technological evolution of interdental brushes combining the benefits of IDB and IRP. IRP eliminate the need for a wire and are therefore without discomfort during insertion. Hence, IRP might be a choice for patients with difficulties using IDB in their daily oral hygiene routine.

However, a recently published meta-review [22] found interdental cleaning with IDB is still the most effective method for interdental plaque removal, and only low evidence for the newer interdental rubber picks exists up to date. Correspondingly, our in vitro results corroborate this meta-review in the fact that IDB are still more effective than IRP.

## Limitations

For our chosen in vitro set-up we have to declare several limitations. As mentioned in our previously investigation [10], in which we described in detail the self-developed experimental setup using powder on resin models to assess ECE and EFC, we are aware that our results could not directly used for extrapolating these data to a clinical situation. For instance, all interdental cleaning aids could only move in a straight direction into the interdental space (Fig. 2). On one side it was a consequence of the technical solution and on the other side it was done for better reproducibility. However, due to space limits and constraints in a patient’s mouth it will be not always possible. Hence, since currently no quantitative, precise, and reproducible method to measure interdental plaque in vivo exists*,* we feel that our experimental set-up is a valid method to measure interdental ECE and EFC with regard to the different anatomies and the interdental spaces and periodontal tissues [10].

## Conclusions

Within the limitations of this in vitro study, experimental cleaning efficacy (ECE) depended on the shape of the interdental areas and was generally best for isosceles triangle shaped interdental areas. Both devices, interdental brushes (IDB) and interdental rubber picks (IRP), demonstrated a positive correlation of cleaning efficacy and force. However, the tested interdental rubber picks currently cannot achieve the high cleaning efficacy of interdental brushes of up to 84%.

## Data Availability

The datasets used and/or analysed during the current study are available from the corresponding author on reasonable request.

## Abbreviations

IDB: Interdental brushes
IRP: Interdental rubber picks
ECE: Experimental cleaning efficacy
ECF: Experimental cleaning force
